# 1-year results after surgery for flexible adult-acquired flatfoot deformity: a cohort study based on 190 patients from the Swedish Foot and Ankle Surgery Register

**DOI:** 10.2340/17453674.2026.45942

**Published:** 2026-05-08

**Authors:** Ida OSBECK, Maria CÖSTER, Isam ATROSHI

**Affiliations:** 1 Department of Clinical Sciences Lund – Orthopedics, Lund University, Lund; 2 Department of Clinical Sciences Malmö - Orthopedics, Lund University, Lund; 3 Department of Surgical Sciences – Uppsala University, Uppsala; 4 Department of Orthopedics Hässleholm-Kristianstad, Skåne University Hospital, Hässleholm, Sweden

## Abstract

**Background and purpose:**

Surgical outcomes in patients with flexible adult-acquired flatfoot deformity (AAFD) have not been evaluated in large prospective register studies. Calcaneal osteotomy and hindfoot arthrodesis are commonly used. We aimed to compare the results of these 2 procedures using preoperative and 1-year postoperative patient-reported outcome data from the Swedish Quality Register for Foot and Ankle Surgery (Swefoot).

**Methods:**

We analyzed data regarding feet that had primary surgery for flexible (grade II) AAFD between 2017 and 2022 in Sweden. The primary outcome was the Self-Reported Foot and Ankle Score (SEFAS), range 0–48 (minimal important difference 5). Secondary outcomes were EQ-5D and satisfaction.

**Results:**

190 feet (63% women, median age 62 years, interquartile range 54–68) were surgically treated during the study period. Mean improvement in SEFAS score was 12 (95% confidence interval [CI] 10–13) in grade IIa and 10 (CI 8–12) in grade IIb, and in the EQ-5D index 0.27 (CI 0.20–0.34) and 0.23 (CI 0.15–0.31), respectively. Compared with preoperatively a higher percentage of patients were satisfied with postoperative foot appearance (77% vs 39%) and foot strength (66% vs 23%). The mean SEFAS score improvement was 11 (CI 10–13) in the osteotomy group and 10 (CI 5–15) in the arthrodesis group; adjusted mean difference was 2.7 (CI –1.2 to 6.5).

**Conclusion:**

Surgical treatment of flexible AAFD significantly improves function and quality of life at 1 year postoperatively. No differences in postoperative patient-reported outcomes were observed between patients who underwent calcaneal osteotomy compared with hindfoot arthrodesis.

Adult acquired flatfoot deformity (AAFD) is a disabling condition with symptoms varying from mild pain along the posterior tibial tendon to severe rigid deformity of the foot and osteoarthritis [1,2]. In patients requiring surgery, different soft-tissue and bone procedures are used, often in combination, partly depending on whether the deformity is flexible or rigid. Using data from the Swedish Quality Register for Foot and Ankle Surgery (Swefoot), we have previously described the surgical interventions used in the treatment of AAFD according to severity. We found that flexible AAFD (grades IIa and IIb) were the most common grades treated surgically and that substantial regional variations existed in the type of surgery. To our knowledge, the outcomes of surgical treatment for flexible AAFD have not been evaluated in population-based studies, and no comparisons have been made regarding the efficacy of different interventions.

The aims of this register-based study are (i) to describe the interventions used and 1-year postoperative patient-reported outcomes in the treatment of flexible AAFD, according to grade, in Sweden and (ii) to compare the results of calcaneal osteotomy and hindfoot arthrodesis.

## Methods

### Study design and inclusion criteria

This prospective register-based cohort study is reported in accordance with the STROBE guidelines. The study is based on data from the Swefoot register. Swefoot comprises both surgeon-reported and patient-reported data, including patient-reported outcome measures implemented by the majority of reporting centers in 2017 [3]. Swefoot validation is performed by comparison with medical records at several major centers across Sweden, as well as through routine data-quality reports and continuous internal monitoring conducted at Registercentrum (a network of regional competence centers that support national quality registers). Completeness is assessed by linkage with the Swedish National Patient Register, and Swefoot currently covers 87% of register-eligible diagnoses; completeness for AAFD is approximately 60%.

In June 2023 we retrieved data on all feet treated surgically for AAFD up to May 2023 from Swefoot. The inclusion criteria for this study were feet that had undergone primary surgery for flexible (grade II) AAFD registered between February 2017 and May 2022. The exclusion criteria were AAFD grade I or III, previous ipsilateral surgery for AAFD, and absence of data concerning the surgery.

### Patient-reported data

The patients completed questionnaires preoperatively at the hospital and postoperatively via a secure website. The questionnaires included demographic information (age, sex, height, weight, and smoking status), medical conditions (diabetes and rheumatic disease), the Self-Reported Foot and Ankle Score (SEFAS) and the 3-level EuroQol 5-dimensions (EQ-5D-3L). The patients also responded to questions regarding their satisfaction with foot appearance, footwear options, and strength of the affected foot (at baseline and 1 year postoperatively) and satisfaction with the results of surgery (at 1 year postoperatively). The questions regarding satisfaction had 5 response options: very satisfied, satisfied, quite satisfied, neither satisfied nor dissatisfied, and dissatisfied; the first 3 responses were grouped as “satisfied” and the last 2 as “not satisfied”.

The SEFAS is a 12-item region-specific PROM in which patients rate function and pain in their foot and ankle and their quality of life [4]. The score ranges from 0 (worst function and pain) to 48 (normal function without pain). The minimally important change (MIC) for hindfoot disorders is 5 points [5]. The SEFAS is validated with good psychometric properties in patients with foot and ankle disorders [6,7]. The EQ-5D index ranges from values less than 0 (worst) to 1 (full health). The patients also estimate their general health on a 0–100 visual analog scale (VAS).

### Surgeon-reported data

Immediately after the surgery, the surgeon reports AAFD grade and type of interventions performed [3]. In this study we present the interventions as various soft-tissue procedures, osteotomies, and arthrodeses. We also compared calcaneal osteotomy with hindfoot arthrodesis, which includes arthrodesis of 1 or more of the talonavicular, talocalcaneal, and calcaneocuboid joints.

### Statistics

The primary outcome was change in SEFAS score from baseline to 1 year postoperatively. When response to a single item was missing, it was substituted with the mean of the remaining 11 items; if responses to 2 or more items were missing, no score was computed. The secondary outcomes were changes in EQ-5D index (calculated using UK Tariff), EQ-5D VAS score, satisfaction with foot appearance, footwear options, and foot strength and satisfaction with the result of surgery at 1 year postoperatively. Changes in SEFAS score, EQ-5D index, and VAS score, according to grade, were analyzed with a paired-samples t-test. The McNemar test was used to analyze change in satisfaction with appearance of the foot, footwear options, and foot strength from preoperatively to 1-year postoperatively. To assess the relationship between surgical procedure (calcaneal osteotomy vs hindfoot arthrodesis) and postoperative SEFAS score we used analysis of covariance (ANCOVA) adjusting for sex, age, BMI, rheumatic disease, baseline score, and concomitant procedures. We calculated least square means with standard errors and 95% confidence interval (CI) and the between-group differences. Because there were 3 individuals with bilateral surgery in the osteotomy group we performed a sensitivity analysis excluding these patients. Handling of missing data is important [8]. With regard to patients in Swefoot with missing responses to the SEFAS score preoperatively or/and postoperatively we assumed that data was missing at random and performed a non-responder analysis. For the covariates included in the ANCOVA comparing SEFAS scores in the osteotomy and arthrodesis groups only 1 patient had missing data and this was not replaced [9]. All statistical tests were 2-sided and a P value < 0.05 was considered statistically significant. We used IBM SPSS Statistics 28 (IBM Corp, Armonk, NY, USA) for statistical analyses.

### Ethics, data sharing plan, funding, use of AI tools, and disclosures

The study was approved by the Swedish Ethical Review Authority (2020-05550, 2020-11-11). Swefoot is approved by the Swedish Data inspection Board and operates in accordance with Swedish and EU data protection regulations. All patients entered in the register are informed about Swefoot, the registration, and that they have the right to decline. According to Swedish legislation, national quality registers do not need signed consent from the individual patient. Data access to researchers other than the researchers performing this study must be granted by the Ethical Review Authority and the register. Swefoot is funded by the Swedish Association of Local Authorities and Regions. We used AI-based language assistance only for rephrasing some sentences. The final content was reviewed and approved by the authors. All authors report no conflict of interests. Complete disclosure of interest forms according to ICMJE are available on the article page, doi: 10.2340/17453674.2026.45942

## Results

**Figure 1:**
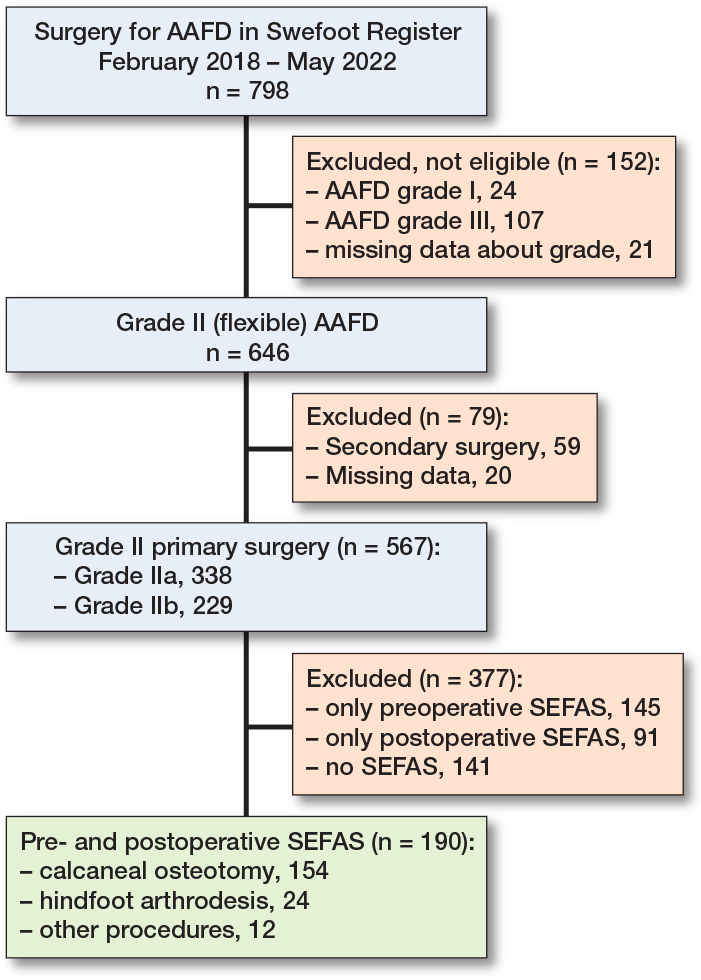
Study flowchart. AAFD: Adult Acquired Flatfoot Deformity; CO: calcaneal osteotomy; HFA: hindfoot arthrodesis; SEFAS: Self-Reported Foot and Ankle Score; Swefoot: National Quality Register for Foot and Ankle Surgery.

**Figure 2:**
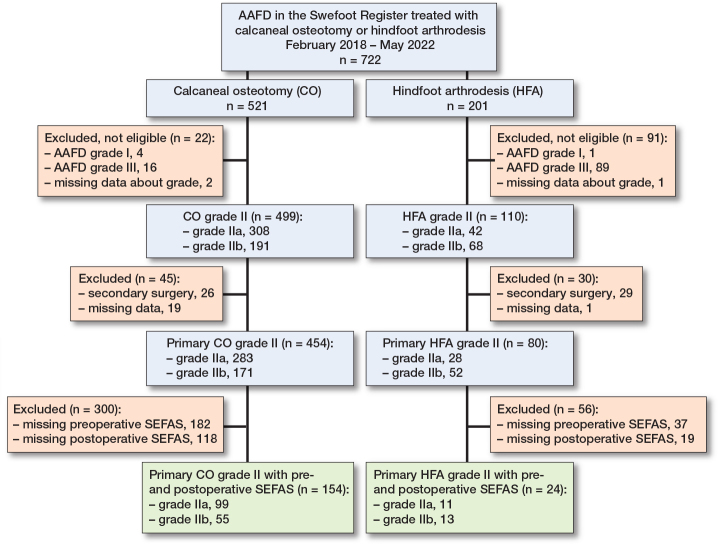
Study flowchart for calcaneal osteotomy and hindfoot arthrodesis. For abbreviations, see Figure 1

Between February 2017 and May 2022, AAFD surgery was registered for 798 feet, of which 567 feet had primary surgery for AAFD grade II (Figure 1). Of these, 190 patients had complete preoperative and postoperative SEFAS scores. In group IIa (n = 119) and group IIb (n = 71), there were 81 (68%) and 38 (54%) women, with median age of 61 (interquartile range [IQR] 54–68) and 62 (IQR 53–69) years, respectively. No important difference, regarding characteristics, was found between patients with grade IIa and IIb (Table 1). Of the 567 feet with grade II, 335 (59%) had preoperative SEFAS score, 281 (50%) had a postoperative SEFAS score, and 190 (34%) had both preoperative and postoperative scores. Patient characteristics of the nonresponders were generally similar to those with complete responses (Supplementary Table 1). Of the 190 patients with preoperative and postoperative SEFAS scores 178 patients had undergone either calcaneal osteotomy (n = 154) or hindfoot arthrodesis (n = 24; including 6 patients with concomitant osteotomy) (Figure 2). No important difference in characteristics was found between the 2 groups (Supplementary Table 2).

**Table 1:** Patient characteristics according to AAFD grade in grade II patients with pre- and postoperative SEFAS scores (N = 190)

Variable	IIa (n = 119)	IIb (n = 71)
Age, median (IQR)	61 (54–68)	62 (53–69)
Sex, n (%)		
Men	38 (32)	33 (47)
Women	81 (68)	38 (54)
BMI, mean (SD)	28.6 (4.7)	28.9 (4.8)
Missing values, n	1	–
Diabetes, n (%)	8 (6.8)	2 (2.8)
Missing values, n	1	–
Rheumatic disease, n (%)	10 (8.5)	7 (9.9)
Missing values, n	1	–
Smoker, n (%)		
No	107 (93)	64 (93)
Yes	3 (2.6)	1 (1.4)
Yes, but preoperative cessation	5 (4.3)	4 (5.8)
Missing values, n	4	2
Side, n (%)		
Right	62 (52)	34 (48)
Left	57 (48)	37 (52)

BMI: body mass index; IQR: interquartile range; SEFAS: Self-Reported Foot and Ankle Score.

### Type of surgical interventions

The most frequently used soft-tissue procedures in both grade IIa and IIb were posterior tibial tendon repair and flexor digitorum longus transfer, being performed in more than 60% of the feet (Table 2). The most common bony procedure was medial displacement calcaneal osteotomy in grade IIa (74%) and lateral column lengthening in grade IIb (51%), while hindfoot arthrodesis was performed in 11% of grade IIa and 13% of grade IIb cases. Other procedures performed in grade IIa included spring ligament repair (34%), first tarsometatarsal joint arthrodesis (16%), and tenosynovectomy (15%). In grade IIb, spring ligament repair was performed in 28%, first tarsometatarsal joint arthrodesis in 24%, and tenosynovectomy in 13%. Achilles tendon or gastrocnemius lengthening was performed in 17% and 35%, respectively (Table 2).

**Table 2:** First-time surgical procedures in the treatment of flexible AAFD, according to grade, in patients with preoperative and postoperative SEFAS score and in all registered patients. Values are count (%)

Procedure	Patients with preoperative and postoperative SEFAS score (n = 190)		All registered patients (n = 567)	
	IIa	IIb	IIa	IIb
	(n = 119)	(n = 71)	(n = 338)	(n = 229)
Soft-tissue procedures				
PTT repair	85 (71)	49 (69)	230 (68)	136 (59)
FDL transfer	71 (60)	43 (61)	209 (62)	142 (62)
SL repair	40 (34)	20 (28)	100 (30)	58 (25)
Tenosynovectomy	18 (15)	9 (13)	55 (16)	28 (12)
AT or GC lengthening	20 (17)	25 (35)	64 (19)	70 (31)
Bone procedures				
MDCO	88 (74)	16 (23)	253 (75)	50 (22)
LCL	5 (4.2)	36 (51)	19 (5.6)	118 (52)
MDCO and LCL	4 (3.4)	34 (48)	17 (5.0)	89 (39)
HFA **^a^**	11 (9.2)	13 (18)	28 (8.3)	52 (23)
HFA + CO	6 (5.0)	14 (20)	2 (0.6)	4 (1.7)
NC arthrodesis	7 (5.9)	6 (8.5)	12 (3.6)	10 (4.3)
TMT-1 arthrodesis	19 (16)	17 (24)	41 (12)	40 (18)

a Arthrodesis of the talocalcaneal, talonavicular, or calcaneocuboid joint, or combination.

AT: Achilles tendon; CO: calcaneal osteotomy; FDL: flexor digitorum longus; GC: gastrocnemius; HFA: hindfoot arthrodesis; LCL: lateral column lengthening; MDCO: medial displacement calcaneal osteotomy; NC: naviculocuneiform; SL: spring ligament; TMT: tarsometatarsal.

The 12 feet that were not treated with osteotomy or arthrodesis had 1 or more of the following procedures: 9 posterior tibial tendon repair, 2 flexor digitorum longus transfer, 2 spring ligament repair, 1 tenosynovectomy, 1 first tarsometatarsal joint arthrodesis, and 1 Achilles tendon lengthening.

No important difference regarding surgical procedures was found comparing the patients who had complete preoperative and postoperative SEFAS scores (n = 190) and all patients with primary surgery for grade II (n = 567) (see Table 2).

### Patient-reported outcomes after surgical treatment of flexible AAFD

The mean preoperative SEFAS score was 18 (SD 7) in both grade IIa and IIb, and the mean change was 12 (CI 10–13) in grade IIa and 10 (CI 8–12) in grade IIb (Table 3). The mean preoperative EQ-5D index was 0.44 (SD 0.32) in grade IIa and IIb, and the mean change was 0.27 (CI 0.20–0.34) in grade IIa and 0.23 (CI 0.15–0.31) in grade IIb. The percentage of patients satisfied with foot appearance increased from 41% preoperatively to 75% at 1 year postoperatively in grade IIa and from 35% preoperatively to 79% at 1 year in grade IIb (Table 4).

**Table 3:** Preoperative, 1-year postoperative, and mean change in patient-reported outcomes according to AAFD grade

PROM	Grade IIa				Grade IIb			
	n	Preoperative	Postoperative	Change	n	Preoperative	Postoperative	Change
		mean (SD)	mean (SD)	mean (CI)		mean (SD)	mean (SD)	mean (CI)
SEFAS score	119	18 (7)	30 (9)	12 (10–13)	71	18 (7)	28 (10)	10 (8–12)
EQ-5D index	116	0.44 (0.32)	0.71 (0.23)	0.27 (0.20–0.34)	68	0.44 (0.32)	0.68 (0.30)	0.23 (0.15–0.31)
EQ-5D VAS	112	61 (24)	73 (19)	12 (8–17)	69	64 (24)	72 (21)	7 (2–12)

CI: 95% confidence interval; EQ-5D: EuroQol 5 Dimensions; SEFAS: Self-Reported Foot and Ankle Score; VAS: visual analog scale.

**Table 4:** Percentage of patients satisfied with foot appearance, shoe wear, and strength preoperatively vs 1-year postoperatively according to AAFD grade. Values are count (%)

Satisfaction	n ^a^	IIa		n ^a^	IIb	
		Preop.	Postop.		Preop.	Postop.
Appearance	126	52 (41)	95 (75)	84	29 (35)	66 (79)
Shoe wear	128	77 (60)	97 (76)	84	40 (48)	69 (82)
Strength	119	22 (19)	77 (65)	75	23 (31)	52 (69)

a Number of patients with preoperative and postoperative response to the satisfaction items.

All comparisons of satisfaction preoperatively vs postoperatively are statistically significant (P < 0.01, McNemar test).

### Patient-reported outcomes after calcaneal osteotomy vs hindfoot arthrodesis in flexible AAFD

The mean preoperative SEFAS score was 19 (SD 7) in the osteotomy group and 16 (SD 6) in the arthrodesis group, and mean score change was 11 (CI 10–13) and 10 (CI 5–15), respectively. The mean changes in SEFAS score, EQ-5D index, and EQ-5D VAS did not differ significantly between the 2 groups (Table 5). In the multivariable ANCOVA analysis, the adjusted mean difference between calcaneal osteotomy and hindfoot arthrodesis was 2.7 (CI –1.2 to 6.5) (Table 6). The sensitivity analysis excluding the 3 patients with bilateral surgery gave similar results.

**Table 5:** Preoperative, 1-year postoperative, and mean change in patient-reported outcomes according to surgical procedure

PROM	Grade IIa				Grade IIb				Unadjusted mean between-group difference in change from baseline (CI)
	n	Preoperative	Postoperative	Change	n	Preoperative	Postoperative	Change	
		mean (SD)	mean (SD)	mean (CI)		mean (SD)	mean (SD)	mean (CI)	
SEFAS score	154	19 (7)	30 (9)	11 (10–13)	24	16 (6)	26 (11)	10 (5 to 15)	1.3 (–2.6 to 5.1)
EQ-5D index	149	0.47 (0.31)	0.71 (0.26)	0.24 (0.18–0.29)	23	0.30 (0.32)	0.64 (0.30)	0.34 (0.16 to 0.51)	–0.10 (–0.26 to 0.06)
EQ-5D VAS	148	63 (23)	73 (20)	10 (7–14)	22	57 (25)	69 (22)	12 (–1 to 24)	–1 (–1 to 9)

a LCL, MDCO or both, without HFA (see Table 2).

b Arthrodesis of the talocalcaneal, talonavicular, or calcaneocuboid joint (includes 1 foot with HFA combined with CO).

For abbreviations, see Tables 2 and 3.

**Table 6:** Multivariable analysis comparing the postoperative SEFAS score in the calcaneal osteotomy and hindfoot arthrodesis groups

Group	n	Least squares mean (CI) ^a^	SE	Difference in means (CI) ^a^	P value
CO	154	29.4 (27.0–31.8)	1.2	_2.7 (–1.2 to 6.5)_	0.2
HFA	24	26.8 (22.9–30.6)	2.0		
Sensitivity analysis **^b^**					
CO	148	29.3 (26.9–31.7)	1.2	_2.3 (–1.5 to 6.1)_	0.2
HFA	24	27.0 (23.1–31.7)	2.0		

a ANCOVA adjusting for sex, age, BMI, rheumatic disease, baseline SEFAS score, and concomitant procedure.

b Same analysis excluding 3 individuals with bilateral surgery in the CO group.

CI: 95% confidence interval; CO: calcaneal osteotomy; HFA: hindfoot arthrodesis; SE: standard error.

## Discussion

This is, to our knowledge, the first study to use a nationwide register to describe patient-reported outcomes in patients treated surgically for AAFD and to compare the results of calcaneal osteotomy and hindfoot arthrodesis.

We evaluated change in patient-reported symptoms and activity limitations in patients surgically treated for AAFD in Sweden. We showed improvement from baseline to 1 year after surgery as measured with SEFAS score, EQ-5D index, and EQ-5D VAS. We also showed improvement in satisfaction with foot appearance and strength from baseline to 1 year after surgery. There were no statistically significant difference in patient-reported outcomes in patients treated with calcaneal osteotomy compared with hindfoot arthrodesis. The improvement in SEFAS score was greater than the previously estimated minimal important change value of 5 units, indicating a clinically important improvement [5]. The reported normal mean values for the SEFAS score among women in the age groups 50–59 and 60–69 years are 42 and 40, and for men in the same age groups are 46 and 44, respectively [6]. Although there was a large overall improvement, no subgroup had a higher mean postoperative SEFAS score than 30 at 1 year postoperatively. Thus, surgical treatment rarely results in full symptom resolution and a normally functioning foot. In a previous study, 21 patients with AAFD had a slightly better mean SEFAS score at 2 years after surgery than the mean score at 1 year shown in our study (32 vs 29) [10]. Considering the long rehabilitation time after both osteotomy and arthrodesis, further improvement may occur after 1 year.

In addition to improved SEFAS scores the percentage of patients satisfied with foot appearance and strength and footwear options was significantly higher at 1 year postoperatively than before surgery.

### Surgical procedures

As shown in our previous study describing the surgical procedures in the treatment of AAFD grade I–III in Sweden [11,12], we observed frequent use of hindfoot arthrodesis for a similar group of patients usually treated with calcaneal osteotomy, despite arthrodesis not usually being the first-line surgical treatment method in flexible AAFD [11,12]. Further, less than a third of the patients were treated with spring ligament repair or Achilles tendon or gastrocnemius lengthening. In our previous study, we also described a low rate of these 2 soft-tissue procedures, but grade II was the grade at which spring ligament repair was most frequently performed [11]. Recent studies have highlighted the importance of the spring ligament in achieving good functional and radiographic outcomes, particularly in maintaining medial peritalar stability, but no studies have compared the outcomes of patients treated with and without spring ligament repair [13,14]. It can be speculated whether surgeons do not contemplate a potentially elongated spring ligament or are less inclined to reconstruct the ligament for technical reasons, and this issue needs further investigation.

Procedures involving the medial column are also common elements in the surgical treatment of AAFD. They are used to both stabilize and desupinate the forefoot. There are different procedures available and first tarsometatarsal joint arthrodesis is one of the more commonly used in Sweden [11]. In this study, the procedure was slightly less common in grade IIa than in grade IIb, an expected finding given that grade IIb is associated with forefoot supination, and possibly increased instability in the medial column. Other stabilizing procedures of the medial column were also used, including opening-wedge medial cuneiform osteotomy (Cotton procedure) and plantar-flexing first metatarsal osteotomy. Because the question regarding whether these 2 procedures were performed during the surgery was first added to the register in 2022, we chose not to include this data.

### Patient-reported outcomes after calcaneal osteotomy vs hindfoot arthrodesis

In both the osteotomy and arthrodesis groups, SEFAS score and EQ-5D index improved significantly. The arthrodesis group had slightly worse preoperative scores than the osteotomy group indicating worse function and quality of life, even though both groups included grades IIa and IIb patients. Although, in the adjusted analysis, the SEFAS score showed a modestly better postoperative score in the calcaneal osteotomy group compared with hindfoot arthrodesis, the difference was not statistically significant.

In a retrospective chart-review study no difference was found between talocalcaneal arthrodesis and lateral column lengthening in postoperative Foot and Ankle Outcome Score; however, no adjustment was made for the significantly worse preoperative score in the arthrodesis group [15].

### Limitations

We had a high rate of missing responses to patient-reported outcome measures. However, the characteristics of patients who had missing responses preoperatively and/or at 1 year postoperatively were similar to those who completed both preoperative and postoperative PROMs. The follow-up time of 1 year is relatively short considering that many surgically treated patients have a long recovery time for postoperative improvement. The rigidity resulting from hindfoot arthrodesis is a symptom that may become a problem detected with longer follow-up.

We had a small sample size of patients with grade II AAFD treated with either calcaneal osteotomy or hindfoot arthrodesis. It may be argued that the results of osteotomy and arthrodesis ought to be analyzed separately in grade IIa and IIb as these grades differ in clinical presentation. Furthermore, patients in the osteotomy group were treated with medial-displacement or/and lateral-column lengthening osteotomy combined with various soft-tissue procedures. The hindfoot arthrodesis group had arthrodesis of 1 or more joints, also combined with various soft-tissue procedures (and 6 patients also underwent concomitant calcaneal osteotomy), which could be a source of confounding. We chose not to stratify our analysis further in order to create a large enough sample size for linear regression modelling.

Although Swefoot has a relatively high coverage of 87%, the completeness of AAFD registration is approximately 60%, which is an important limitation [16]. The low registration rates during the initial years continue to impact current figures, despite the fact that completeness improves every year. Achieving high completeness of PROM collection is frequently limited by logistic challenges in register-based data collection. These include variability in follow-up methods, reliance on different platforms (paper, electronic, telephone), and diverse clinical workflows across institutions. As a result, the frequency and consistency of patient responses vary substantially [17]. Recognizing these difficulties, the International Society of Arthroplasty Registries Working Group has set a realistic completeness threshold of 60% in arthroplasty registers [18].

### Strengths

A major strength of this study is the use of register-based data, which reflects real-world clinical practice and includes a large number of surgeons and surgical centers. While surgical outcomes may be influenced by differences in techniques or surgeon experience, such data enable longitudinal monitoring of treatment outcomes and provide a comprehensive picture of routine care across regions.

### Conclusion

Surgical treatment of flexible AAFD significantly improves function and quality of life at 1 year postoperatively. No differences in postoperative patient-reported outcomes were observed between patients who underwent calcaneal osteotomy compared with hindfoot arthrodesis.

*In perspective,* we have not found any other study comparing the patient-reported outcomes after hindfoot arthrodesis and calcaneal osteotomy in the treatment of flexible AAFD. Current guidelines do not recommend hindfoot arthrodesis in the treatment of flexible flatfoot. Considering the potential rigidity associated with hindfoot arthrodesis, a longer follow-up time may reveal larger difference in outcomes between the 2 interventions with regard to function and QoL.

### Supplementary data

Supplementary Tables 1 and 2 are available as Supplementary data on the article home page, doi: 10.2340/17453674.2026.45942

## Supplementary Material



## Notes

IA, MC, and IO conceived the study. All authors contributed to the planning and setting up of the study. Data extraction was conducted by IO. Data analysis was carried out by IO. The manuscript was written by IO with feedback and input from IA and MC.

The authors thank statistician Kaja Doupona Stigsson, Clinical Studies Sweden Forum South for the statistical analysis.

Handling co-editors: Ivan Hvid and Robin Christensen

*Acta* thanks Nikolaus Boehler and Jan Willem Louwerens for help with peer review of this manuscript.
