# Supplementary material for: 1-year results after surgery for flexible adult-acquired flatfoot deformity: a cohort study based on 190 patients from the Swedish Foot and Ankle Surgery Register
Source: Acta Orthop. 2026 May 8;97:279–85. doi: 10.2340/17453674.2026.45942 (PMC13154001; doi:10.2340/17453674.2026.45942)
Supplement: Supplementary file 1 [file ActaO-97-45942-s1.pdf]

Supplemental Table 1. Characteristics of the patients according to missing SEFAS scores

	Only preoperative SEFAS score		Only postoperative SEFAS score		No SEFAS score	
	IIa (n = 77)	IIb (n = 68)	IIa (n = 61)	IIb (n =3 0)	IIa (n = 81)	IIb (n = 60)
Age, median	59	57	60	62	57	61
(IQR)	(46–64)	(51–67)	(53–70)	(54–69)	(50–67)	(53–68)
Sex, n (%)						
Men	24 (31)	28 (41)	12 (20)	12 (40)	24 (30)	25 (42)
Women	53 (69)	40 (59)	49 (80)	18 (60)	57 (70)	35 (58)
BMI, mean (SD)	27.8 (4.4)	28.0 (4.2)	-	-	-	-
Diabetes, n (%)	1 (1.3)	6 (8.8)	-	-	-	-
Rheumatic disease, n (%)	7 (9.1)	7 (10)	-	-	-	-
Side, n (%)						
Right	31 (40)	29 (43)	34 (56)	19 (63)	38 (47)	28 (47)
Left	46 (60)	39 (57)	27 (44)	11 (37)	43 (53)	32 (53)
SEFAS score, mean (SD)						
Preoperative	19 (8)	18 (8)	-	-	-	-
1-year postoperative			29 (11)	31 (10)		

SEFAS, Self-reported Foot and Ankle Score

Supplemental Table 2. Characteristics of the patients treated with calcaneal osteotomy (CO) or hindfoot arthrodesis (HFA)

Characteristic	CO (n = 154)	HFA (n = 24)
Age, median (IQR)	61 (54–68)	67 (60–72)
Sex, n (%)		
Men	62 (40)	5 (21)
Women	92 (60)	19 (79)
BMI, mean (SD)	28.9 (4.7)	29.7 (5.0)
Missing	9	–
Diabetes, n (%)	9 (5.8)	1 (4)
Missing	1	–
Rheumatic disease, n (%)	16 (11)	4 (17)
Missing	4	–
Smoker, n (%)		
No	138 (92)	23 (100)
Yes	4 (2.7)	0 (0)
Yes, preoperative cessation	8 (5.3)	0 (0)
Missing	4	1
Side, n (%)		
Right	78 (51)	12 (50)
Left	76 (49)	12 (50)

IQR, interquartile range
